# Increased pulmonary embolism in patients with COVID-19: a case series and literature review

**DOI:** 10.1186/s40794-021-00145-3

**Published:** 2021-06-12

**Authors:** Sonia Hesam-Shariati, Poya Fatehi, Morteza Abouzaripour, Fardin Fathi, Negin Hesam-Shariati, Mohammad Bakhtiar Hesam Shariati

**Affiliations:** 1 School of Medical Sciences, Faculty of Medicine, University of New South Wales, Sydney, Australia; 2 Department of Radiology, Tohid Hospital, Kurdistan University of Medical Sciences, Sanandaj, Iran; 3 Department of Anatomical Sciences, Faculty of Medicine, Kurdistan University of Medical Sciences, Sanandaj, Iran; 4 Cellular and Molecular Research Center, Research Institute for Health Development, Kurdistan University of Medical Sciences, Sanandaj, Iran

**Keywords:** COVID-19, Venous thromboembolism, Pulmonary embolism, Computed tomography angiography

## Abstract

There is some recent evidence that the coronavirus disease 2019 (COVID-19) increases the risk of venous thromboembolism by creating a prothrombotic state. COVID-19 and pulmonary embolism (PE) are both associated with tachypnoea, hypoxemia, dyspnoea, and increased D-dimer. Diagnosis of pulmonary embolism in a patient with COVID-19 compared to an individual without it, using the conventional clinical and biochemical evidence is challenging and somehow impossible. In this study, we reported four male cases affected by COVID-19 and admitted to hospitals in Sanandaj, Iran. The patients were all older adults (ranging between 56 and 95 years of age). Fever, chills, muscle pain, and cough were evident in all the cases. Red blood cell levels were low, and pulmonary embolism was clearly detected on spiral computed tomographic (CT) angiography of the pulmonary circulation of all patients. These cases demonstrated that COVID-19 may lead to pulmonary embolism by causing blood coagulation problems. As COVID-19 continues to cause considerable mortality, more information is emerging which reveals its complicated pathogenicity. In the meantime, venous thromboembolism remains an uncommon finding in patients with COVID-19. It is essential that health care providers perform the necessary diagnostic evaluations and provide appropriate treatment for patients.

## Introduction

Coronaviruses are a large family of enveloped ribonucleic acid (RNA) viruses found in animals such as pigs, camels, bats, and cats. Entering such viruses in the human body can cause mild to moderate upper respiratory illnesses [1]. Coronavirus disease 2019 (COVID-19) or acute coronavirus syndrome 2 (SARS-CoV-2), caused by the new coronavirus, usually appears with mild symptoms; however, in 14% of cases, it can lead to a severe illness that requires hospitalization [2]. Severe hypoxemia is the main characteristic of the severity of this disease [3]. To this date, COVID-19 has infected more than 150 million people worldwide, with over 3 million deaths [4]. Studies have shown that COVID-19 infection increases the risk of venous thromboembolism (VTE) in patients who are suffering from an increased disseminated intravascular coagulation, inflammation, hypoxemia, and immobility [5, 6]. The incidence rate of VTE in COVID-19 is still unknown. However, emerging data show an increased incidence of venous thromboembolism in COVID-19, especially in more severe cases [7].

So far, there have been several reports of coagulation in patients with COVID-19 [8–10] . It has been suggested that vascular endotheliitis due to an activated immune response or an infection of the vascular endothelium with COVID-19 may lead to blood clotting [11]. Nevertheless, the pathophysiology of coagulation associated with coronavirus is not yet well understood. The incidence of pulmonary embolism (PE) in COVID-19 patients has been reported in many countries, mainly in Europe and the United States [12]. Unfortunately, due to the lack of large prospective studies, there is little information on the epidemiology and pathophysiological mechanisms of COVID-19-associated PE. Timely understanding of these mechanisms is extremely important for the proper diagnosis and management of the deadly complications of PE. In addition, proper dosage and duration of prophylactic anticoagulation are the main concerns for controlling this disease [13, 14]. In this article, we have reported four cases of coronavirus patients with pulmonary embolism admitted to hospitals in Sanandaj, Iran.

## Case 1

**Fig. 1:**
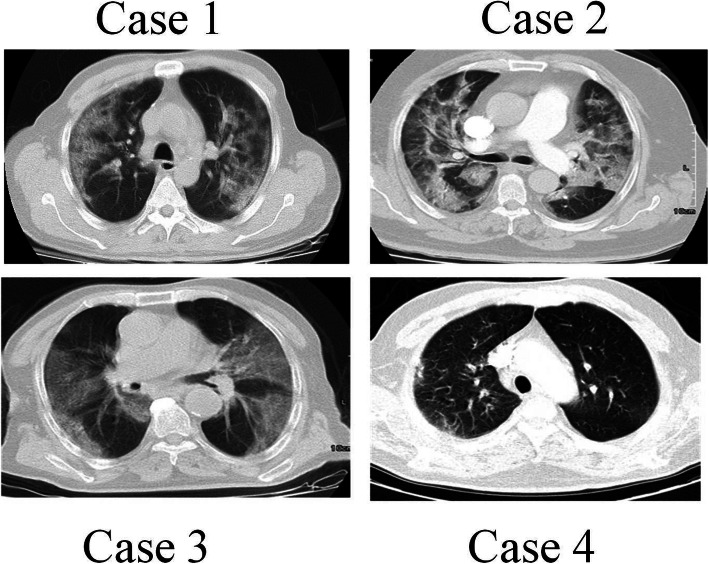
Axial non-contrast-enhanced image of computed tomography (CT) scan of the chest, showing the COVID-19 infection

**Fig. 2:**
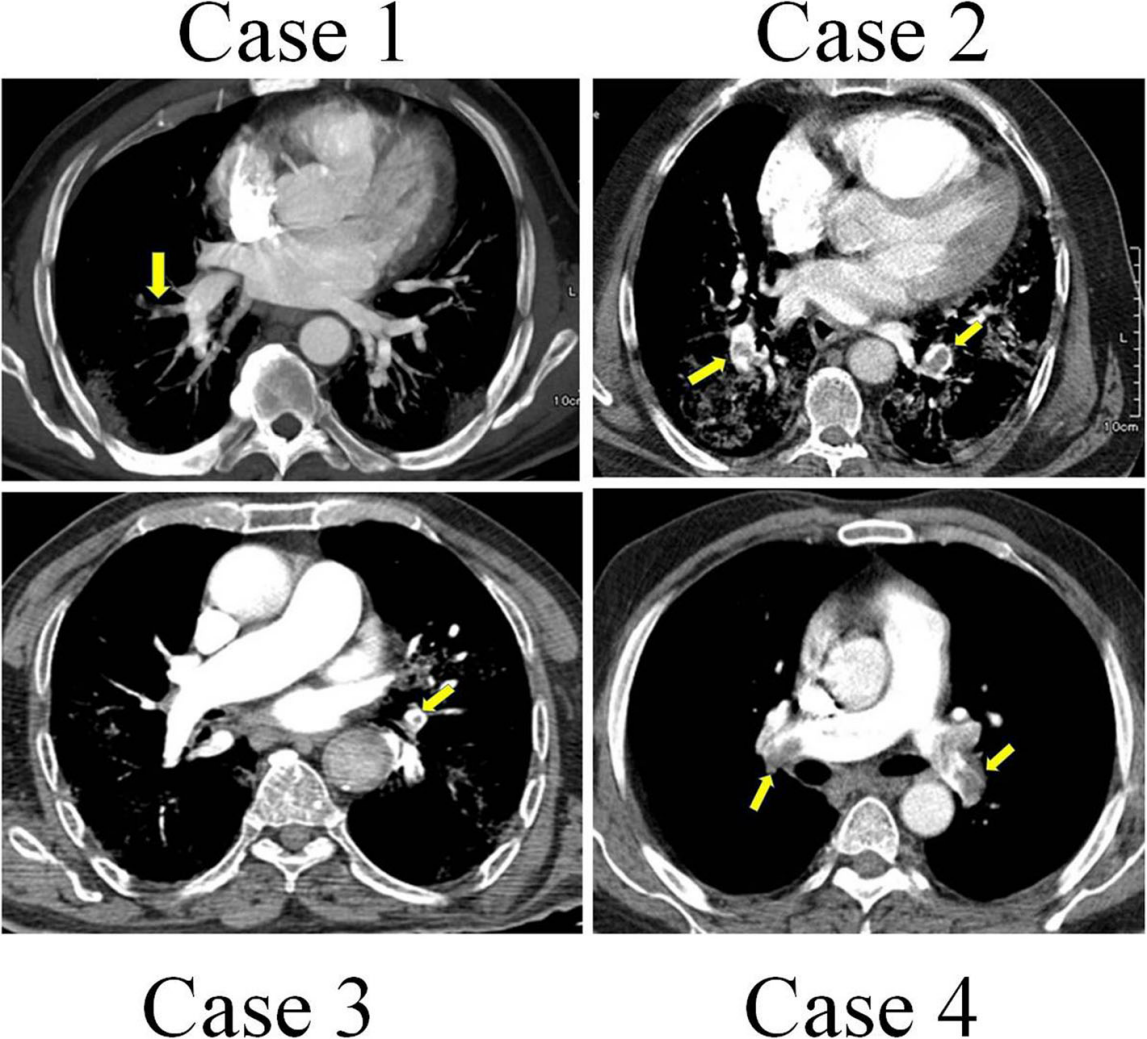
CT angiography images of pulmonary embolism, showing pulmonary artery embolism

A 60-year-old man was presented to the medical unit in July 2020 with symptoms of respiratory problems, severe headache, cough, dizziness, and frequent vomiting. Initial physical and clinical examinations of the patient were normal and there was no underlying disease. The patient had no history of alcohol or tobacco use and was not taking any specific medications at the time. His blood pressure was 120/80 mmHg with a regular pulse rate of 112 beats/min, a respiratory rate of 22 cycles/min, and a temperature of 36 °C. While the patient had no symptoms of arrhythmia, he had mild hypoxemia with an oxygen level of 85–92% (Table 1). Important laboratory findings of the patient are listed in Table 2. PCR on the nasopharyngeal swab sample was performed on the day of hospitalization, which confirmed the diagnosis of COVID-19. The patient was discharged from the hospital after 2 days because his symptoms were relatively mild and there were no other serious symptoms. He was admitted to the hospital 5 days later with respiratory problems, and initial examinations revealed that his oxygen saturation was then 82% on room air. The patient underwent high-resolution computed tomography (CT) scans of the lungs and CT pulmonary angiography. CT scans of the lungs (Fig. 1) showed several diffuse areas of opacity in both right and left lungs, which could indicate viral pneumonia. In addition, on CT angiography of the lungs (Fig. 2), several filling defects were visible in the branch of the pulmonary artery leading to the lower lobe of the right lung, which may indicate acute pulmonary embolism. The patient was started on medications including naproxen to control the muscle pain, hydroxychloroquine and famotidine with antiviral effects, zinc to boost the immune system and repair lung tissue, and neurobion to strengthen the immune system. Some anticoagulant including injected heparin and acetylsalicylic acid tablets, and high-flow oxygen were also used. The severity of COVID-19 in this patient was moderate and he did not require mechanical ventilation or intensive care unit (ICU) management and was released from the hospital 15 days after partial recovery.

**Table 1:** Summary of clinical and medical findings and demographic characteristics of all cases

Characteristics	Unit	Case 1	Case 2	Case 3	Case 4
**Age**	Year	60	56	95	72
**Gender**	Male/ Female	Male	Male	Male	Male
**Primary symptoms**		Respiratory problems Severe headache Cough Dizziness Frequent vomiting	Fever Chills Muscle pain Weakness Cough Tachycardia Acute respirator Syndrome	Fever Cough Diarrhea Chest pain	Fever Cough Weakness Palpitation Respiratory problems
**Baseline medical history**		None	None	Low blood pressure Hyperlipidemia	None
**RT-PCR result**	Negative/ Positive	Positive	Positive	Positive	Positive
**Hospitalization**	Days	15 /Released from the hospital	18/ Released from the hospital	15/ Died	12/ Released from the hospital
**Alcohol or tobacco intake**		No	No	No	–
**Oxygen level in blood**	% in room air	82	84	76	87
**Temperature**	°C	36	39 High	38.5 High	39High
**Heart rate**	beats per minute	112	109	88	–
**Respiratory rate**	cycles/ minute	22	28	60	23
**Median arterial pressure**	mmHg	120/80	130/90	100/65	95/62
**Illness severity**	Mild/ Moderate/ Severe	Moderate	Moderate	Sever	Moderate

**Table 2:** The results of laboratory findings

Test Name	Unit	Reference Range	Case 1	Case 2	Case 3
**BUN**	mg/dl	7–16.8	24 Hi	28 Hi	20
**Ca**	mg/dl	8.6–10.3	8.5 Low	–	–
**p**	mg/dl	2.7–4.5	4.9 Hi	–	–
**Na (ser)**	mEq/L	138–145	139	139	140
**K (ser)**	mEq/L	3.6–5.9	4.3	4.3	3.7
**MCH**	pg	27.5–33.2	28.2	28.2	32.5
**MCHC**	g/dL	30.0–38.0	34.6	31.6	32.6
**Plt**	× 1000/μL	140–440	252	167	72
**Cr**	mg/dl	male:0.8–1.3 mg/dl	1	1.1	1.4 Hi
**SGOT (AST)**	IU/L	Male:< 40	86 Hi	109 Hi	–
**SGPT (ALT)**	IU/L	Male:< 45	46 Hi	55 Hi	–
**WBC**	×1000/μL	4.4–11	3.5 Low	8.6	8
**RBC**	×1,000,000/μL	male:4.5–6.5	4.25 Low	4.04 Low	4.19 Low
**Hb**	g/dl	male:14–17	12 Low	14.2	13.6 Low
**Hct**	%	male:41.5–50.4	34.7 Low	45.0	41.7
**MCV**	fl	80–96	81.6	89.3	99.5
**ALK.P**	IU/L	Male:0–270	617 Hi	280 Hi	–
**CRP**	mg/L	0–6	–	65 Hi	–
**ESR**	mm	5–12	–	55 Hi	–
**D-Dimer**	ng/ml	Normal< 200	–	< 200	–
**CPK**	IU/L	Male:0–171	617 Hi	–	350 Hi
**LDH**	U/L	Male:235–470	810 Hi	–	650 Hi
**Troponin 1**			Negative	–	Negative
**BS**	mg/dl		126	–	127
**P.T.T**	sec	26–38 s	29	–	30
**PT**		11–13 s	13.6	–	15
**INR**			1.2	–	1.6
**PT Control**	%		12	–	11
**Amylase**	IU/L	< 100 U/L	98	–	–
**HBSAg CLIA**	MIU/ml	< 1 non reactive> = 1 reactive	Negative	Negative	Negative
**HCV-Ab CLIA**	MIU/ml	< 1 non reactive> = 1 reactive	Negative	Negative	Negative
**HIV-Ab CLIA**	MIU/ml	< 1 non reactive> = 1 reactive	Negative	Negative	Negative

## Case 2

A 56-year-old man was hospitalized in November 2020 due to the persistence of high fever that started 5 days before. In physical and clinical examinations of the patient, symptoms such as fever, chills, muscle pain, weakness, cough, tachycardia, and acute respiratory syndrome were reported, while there was no report of underlying disease in the patient’s file. Investigations showed no history of alcohol and tobacco intake or any particular medication use at the time. A polymerase chain reaction (PCR)-based test for SARS-CoV-2 was done, and he was diagnosed with COVID-19. Initial examinations of the patient in the hospital revealed a blood pressure of 130/90 mmHg with a regular pulse rate of 109 beats/min, a respiratory rate of 28 cycles/min, and a temperature of 39 °C (Table 1). All laboratory findings of the patient are presented in Table 2. The patient’s electrocardiography (ECG) was normal. There were no changes in the patient’s hemodynamics or respiratory status (oxygen saturation: 84% on room air), and in later stages, due to persistent respiratory problems, CT pulmonary angiography of the patient was ordered by a pulmonologist for further examination. As in CT angiography (Figs. 1 and 2): (a) the diameters of the main pulmonary arteries were normal; (b) defective filling in the lobar, segmental, and sub-segmental branches of the upper, middle, and lower lobes was evident in both lungs, which may indicate thrombosis (embolism); and (c) multiple confluent patchy ground-glass opacification (GGO) and consolidation was found in both lungs. Due to the positive result of COVID-19 test and all the other symptoms mentioned above, the COVID-19 diagnosis was confirmed with a moderate severity. No deep vein thrombosis or other thrombosis were detected. Continuous heparin injection was performed according to the doctor’s instructions to treat pulmonary embolism. Supportive care, antibiotics, and other treatments were used to treat the patient, and he eventually was discharged after 18 days of hospitalization.

## Case 3

A 95-year-old male patient with a history of severe dyspnea for 2 days was referred to a medical center in November 2020. Examination of the patient’s file showed that he had experienced several episodes of diarrhea a few days prior to the onset of shortness of breath, which resolved on its own. According to the patient’s history, his wife had contracted coronavirus 2 weeks before. The examinations also showed that the patient had symptoms of fever, cough, and chest pain. His previous medical history showed low blood pressure and hyperlipidemia, but no history of malignancy. Examination of the family history revealed no signs of coagulation disorders or thromboembolism. The patient never smoked or consumed alcohol. His vital signs included blood pressure of 100/65 mmHg, pulse rate of 88 beats/min, respiratory rate of 60/min, oximetry 76% on room air, and a temperature of 38.5 °C (Table 1). There was no lower extremity edema or calf tenderness. The most common blood and electrolyte tests are listed in Table 2. Testing for viral diseases, including influenza was negative. The initial results of the coronavirus test were negative, but in the second test, which was taken from back of the throat, the coronavirus was detected. According to the patient’s respiratory rate and old age, a CT scan of the lung was performed on the order of the treating physician, and CT images showed a specific sign of the virus (Fig. 1). Due to the deterioration of the patient and having respiratory problems, he was transferred to the hospital’s ICU, approximately 4 days after hospitalization. CT pulmonary angiography was also performed by the order of a pulmonologist. As shown in the CT scan images of the patient, there is a defect of contrast material in the middle lobe branch of the left pulmonary artery, which can indicate chronic thrombosis in this artery (Fig. 2). The patient was started on naproxen, hydroxychloroquine, famotidine, zinc, neurobion, and anticoagulant treatments. Unfortunately, his COVID-19 was severe, and he died due to respiratory failure and intubation after 15 days of hospitalization in the ICU.

## Case 4

A 72-year-old man was hospitalized in a hospital in Sanandaj, Iran in December 2020 after 11 days of cough, fever, weakness, palpitation, and respiratory problems, but no chest pain. The COVID-19 nucleic acid diagnostic test was positive before hospitalization. The severity of his disease was categorized as moderate and the patient was transferred to the quarantine ward of the hospital. At the time of admission, his clinical and physical examination did not show any irregularity in heartbeat, which could be a sign of atrial fibrillation. Other vital symptoms were blood pressure of 95/62 mmHg, temperature of 39 °C, respiration rate of 23 cycles/min, and oxygen saturation rate of 87%. After admission, the laboratory test of COVID-19 was re-confirmed in the hospital (Table 1). Unfortunately, we did not have access to the results of the routine blood tests and the patient’s serum biochemical analyses. Due to respiratory problems, CT angiography of the pulmonary arteries was ordered by his doctor. A defect of filling in the right and left main pulmonary arteries as well as the lobar and segmental branches of both sides was quite obvious (Figs. 1 and 2), which suggested pulmonary embolism. Examination of the lung parenchyma tissue also revealed multifocal turbidity in both lungs, which indicated infection. According to these findings, antiviral, antibacterial, anticoagulant, symptomatic and supportive treatments were started for the patient. Fortunately, with proper and timely treatment, the patient was discharged from the hospital 12 days after admission and the rest of the treatment was continued at home.

## Discussion

Four coronavirus patients with pulmonary embolism were reported in this study. Patients were all from older age range. Indeed, older age has previously been associated with more severe forms of COVID-19 [15]. Patients with COVID-19 often have respiratory symptoms, which make it hard to distinguish from pulmonary embolism in severe cases. Studies have shown that coronaviruses increase the risk of arterial and venous thromboembolism by causing inflammatory reactions, immobility, hypoxia, and disseminated intravascular coagulation (DIC) in patients [16]. The co-occurrence and clinical symptomatic overlaps between pulmonary embolism and COVID-19 has made the diagnosis and treatment of PE more difficult. The presence of COVID-19 pneumonia can be easily detected with RT-PCR and CT scan [17, 18]. However, it is much more difficult to confirm PE; the reason for this is that factors influencing pro-inflammatory and hypercoagulability processes such as lactate dehydrogenase, ferritin, C-reactive protein, and interleukin levels are also increased in patients with coronavirus [19, 20]. In addition, recent studies have shown that levels of D-dimer, fibrinogen, and fibrin degradation products increase in patients with COVID-19 [21]. It has also been shown that even in the absence of pulmonary embolism in patients with COVID-19, the level of D-dimer increases [20]. An increase in D-dimer (> 1 mg/dL) is not a reliable indicator of venous thromboembolism [6, 22], although it may result in mortality. As a result, CT angiography can be helpful in diagnosing VTE in patients with coronavirus [23].

Developing PE have been associated with several risk factors including hypertension, coronary heart disease, malignancy, etc. [24]. Examining medical history of the patients in this study did not show any specific risk factors leading to PE (Table 1). However, it is noteworthy that all four cases in this report were individuals in an older age range (Table 1), which not only increases the risk of developing PE [24], but also leads to more severe forms of COVID-19 [15]. In general, investigating underlying diseases in detail can be useful in better understanding the causal factors of developing PE in patients with COVID-19.

The coagulation mechanism in COVID-19 is unknown. Some theories introduce cytokines as possible factors in the coagulation process in this disease, while others believe that hepatic dysfunction may be involved [25]. Regardless of the coagulation mechanism in patients with coronavirus, it is known that the incidence of thrombosis increases in these patients. This coagulation often extends to intravascular coagulation and this expansion results in venous and arterial thrombosis. In addition, it has been shown that 71.4% of patients who died of COVID-19 met the criteria for diffuse intravascular coagulation [26]. Many patients with COVID-19 face sepsis and septic shock [27]. In the septic process, DIC is a major cause of organ dysfunction, so undergoing anticoagulant therapy in this situation can be very challenging [28].

Although, currently, there are no specific criteria for the use of anticoagulants in COVID-19 patients, heparin or/and other anticoagulants were prescribed for all the reported cases in this study according to an approval from the ministry of health in Iran. However, more clinical trials are needed to determine whether all patients with coronavirus need to be treated with anticoagulants. In general at this point, using PE prophylaxis based on clinical manifestations and D-dimer level, even in mild cases of COVID-19 seems to be important and necessary [12].

Due to insufficient information and different complications in patients with COVID-19, using or not using anticoagulants to improve the overall symptoms of the disease is still highly controversial [29]. Two recent published studies by Klok et al. and Middeldorp et al. advised against prophylactically initiating treatment-dose anticoagulation in all patients with COVID-19 and in opposite, recommended using a lower threshold for proper diagnostic tests in assessing thrombotic complications including deep vein thrombosis and PE [30, 31].

Overall, two aims are recommended to be considered in the treatment of patients with COVID-19. The first goal is to protect the organs and timely diagnosis of events caused by the disease, which can be done by examining blood clot through D-dimer test or ultrasound. The second goal is to strengthen the immune system to prevent the formation of cytokine storms and blood clotting. For this purpose, norobion, zinc and vitamins are widely prescribed for patients with coronavirus in Iran, which were also used for all cases in this study. Further research into clinical trials is needed to clarify whether prophylactic treatment with anticoagulants leads to clinically beneficial outcomes in patients with COVID-19 infection.

## Conclusion

Coronavirus continues to results in significant complications, while more data are emerging that could help to investigate the effects of this disease. The little emerging information suggests the role of coronavirus in increasing systemic coagulation activation, which generally leads to thromboembolic complications such as pulmonary embolism. Here, we presented four rare and notable cases of pulmonary embolism. However, larger studies with wider demographic characteristics are needed to find any possible reason in developing PE in COVID-19 patients. Early and timely diagnosis and treatment of COVID-19 and its complications can be a useful prognosis. Furthermore, studies have shown that this virus can weaken and destroy the immune system by causing pathogenic infections and the associated complications in the body. Thus, strengthening the immune system is one of the most important ways of fighting this disease. Further research is needed on the role of prophylactic anticoagulants in COVID-19 patients and the pathogenesis of hypercoagulability in this disease.

## Data Availability

The patients’ data are presented in Tables 1 and 2, and Figs. 1 and 2 of the manuscript.
